# Overlooked Trends in Observed Global Annual Precipitation Reveal Underestimated Risks

**DOI:** 10.1038/s41598-018-34993-5

**Published:** 2018-11-13

**Authors:** Anne M. Lausier, Shaleen Jain

**Affiliations:** Department of Civil and Environmental Engineering, University of Maine, Orono, ME USA

## Abstract

Numerous human and environmental systems are sensitive to the spatial and temporal distribution of precipitation, including agriculture, water supply, and ecosystems. Trends in observed precipitation form an important line of evidence to understand how changes may increase system vulnerabilities. Linear trends reported in US and global climate assessments reflect changes in mean annual precipitation. Mean trends may not reflect changes across other quantiles in the precipitation probability distribution, including the tails (very high and low precipitation levels), leading to systematic mischaracterization of climate risk. Here we reanalyze global annual precipitation using quantile regression to reveal overlooked trends. We find trends in the tails inconsistent with the mean in 44.4% of land area and 40.7% of rainfed agricultural regions. Previously undetected trends offer a more accurate view of the changing climate. This work enables reappraisals of risk aggregated over thresholds in human and environmental systems, enabling revaluation of threats and identification of appropriate adaptation strategies.

## Introduction

Precipitation is the primary driver of freshwater availability. Portraits of estimated and projected climatic change routinely drive public policy conversations regarding global change and impacts, with precipitation being one of the most prominent variables considered^1,2^. Numerous systems such as agriculture^3,4^, energy systems^5^, water supply^6^, ecosystems^7^, and disease epidemics^8^ show sensitive dependence to precipitation. For example, more than one billion people rely on agriculture as their primary source of livelihood, with over two-thirds of the population of Sub-Saharan Africa involved in agriculture-related activities^9^. When precipitation exceeds thresholds^10^ important to human and environmental systems, both from inter-annual variability and multi-year extremes, the risk of experiencing detrimental impacts increases. Long-term and episodic precipitation excesses and deficits at crop-specific thresholds have been shown to reduce yields^3,11^, and can have significant economic impacts. In one instance, the 2012 US drought event had estimated losses totaling $32.4 billion, largely in the agricultural sector due to low precipitation and high evaporation rates^12^. Changes at one ecologically important threshold can result in a range of interrelated impacts to the system. For example, maintaining forest moisture above biophysical thresholds minimizes system vulnerability to forest fires, disease, and other disturbances that can lead to downstream impacts to populations^13^. Trends at precipitation thresholds for these various contexts may also depart from mean trends, further underscoring the need for flexible approaches to characterize precipitation variability and change.

## Trend Assessment Over the Entire Range of Variability

Historical annual and seasonal trends of precipitation have been extensively studied at regional and global scales^14–18^, as have comparisons with reanalysis data^19,20^ and projections under future conditions^21–23^. The historical record of annual precipitation can be summarized by the probability distribution function (PDF), where the frequency with which precipitation amounts within a certain interval occur and the probability of exceedance (non-exceedance) above (below) a given threshold can be readily estimated. Regression analyses of historical precipitation allow assessments of PDFs over time that aid assessment of: a) threshold exceedances and b) impacts across precipitation-sensitive systems. US^23^ and global climate assessments^21^ have reported annual trends derived from linear regression-based (LR) methods, constituting a baseline for understanding changes in climate risk. However, LR assumes a PDF location-shift model where a) trends are expressed as changes in mean, thus causing the entire PDF to shift and b) variance remains unchanged. Therefore, LR assesses for symmetric changes in precipitation, assuming that increased probability of high (low) annual totals must coincide with a decreased probability of low (high) annual totals. To this end, quantile regression (QR)^24^ is used to assess overlooked trends in annual total precipitation^25^ and overcomes the limits imposed by LR as it a) comprehensively quantifies trends across all thresholds of the PDF, where quantile *τ* corresponds to the precipitation level at which *τ* proportion of the historical data is exceeded, b) does not make distributional assumptions, and c) estimates responses at each *τ* locally, allowing for asymmetric responses across the distribution. While non-parametric methods are flexible, they are limited in their ability to characterize the entire PDF. Previous studies have assessed historical trends in annual-scale extremes^15,26,27^. Trends in these extremes are not necessarily equivalent to the trend in annual totals, leading to a mischaracterization of annual trends. Annual trends accentuate or minimize the impacts of episodic extremes^28^; a reappraisal of annual trends at select thresholds can offer improved understanding of inter-annual precipitation variability and risk.

## Global Reassessment of Precipitation Trends at Specified Thresholds

**Figure 1:**
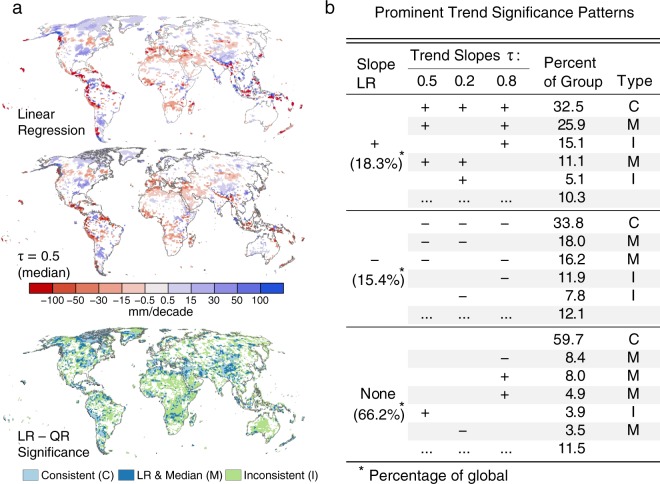
Tail trends show inconsistencies with mean and median. (**a**) Spatial distribution of trends estimated by linear regression (LR) and quantile regression (QR) at *τ* = 0.5 (median). LR-QR significance identifies locations where *τ* = 0.2, 0.5, 0.8 are all consistent in significance and direction with LR (C), where *τ* = 0.5 is consistent with LR (M), and where the responses are inconsistent with LR (I). (**b**) Patterns of QR trends grouped by LR, with consistent responses shown in bold. + and − indicate positive and negative trends respectively and are significant at α = 0.05.

**Figure 2:**
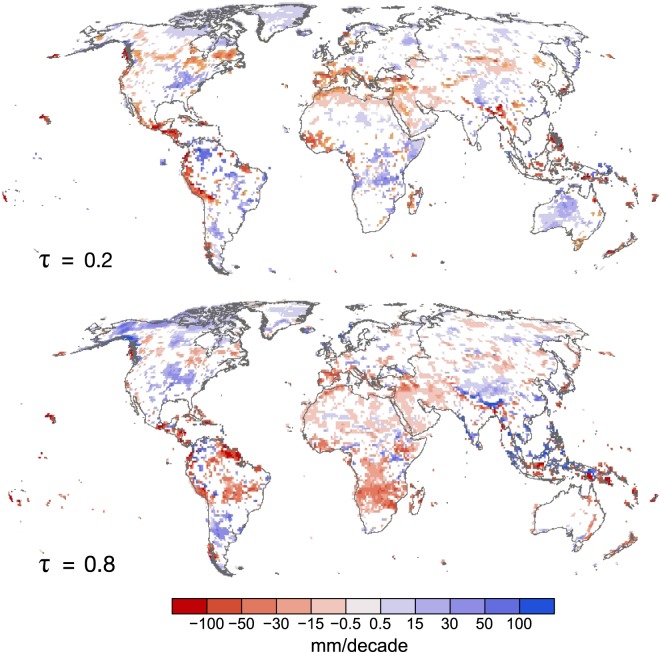
Trends in tails show asymmetries in sign, magnitude, and spatial distribution. Trends in *τ* = 0.2 and *τ* = 0.8 estimated by QR, and are significant at 𝛼 = 0.05

Trends in the median (*τ* = 0.5) (Fig. 1a) and the dry (*τ* = 0.2) and wet (*τ* = 0.8) tails of precipitation PDF (very low and high precipitation levels) (Fig. 2), are used to characterize annual total precipitation variability on the global scale. *τ* = 0.2 and *τ* = 0.8 are selected as the representative thresholds for dry and wet tails of the precipitation distribution, respectively. Given the modest sample size, more extreme quantiles (*τ* > 0.8 and *τ* < 0.2), while calculable, are associated with high uncertainty (Supplementary Fig. S1). Trends in LR and τ = 0.5 are consistent in most locations as they are both measures of central tendency, with *τ* = 0.5 a more robust measure due to its insensitivity to outliers. Considering the location-shift model whereby a significant trend in LR corresponds to a significant trend in the same direction for all quantiles evaluated, we intersect the direction and significance of trends in LR and QR (Fig. 1a). Three possible patterns are assumed by the location-shift model: LR and all quantiles are consistent in significance and direction of trend (C), only LR and the median are consistent (M), and LR and quantiles are inconsistent (I). We find consistencies in significant trends across LR and all quantiles in the eastern United States, western South America, northern latitudes of North America, and dispersed throughout areas of Asia comprising 11.2% of all grid cells. An additional 13.4% of grids show consistencies between LR and the median only. However, we find that there are asymmetric trends in all regions with 35.9% of total grids showing inconsistencies between LR and quantiles in significance, direction, or both. Comparing the primary patterns of QR and LR trends at each location highlights the diversity of QR assessed trends, including the 3 possible patterns assumed by a location-shift model (Fig. 1b, Supplementary Table S1). We find that the tails consistently exhibit asymmetric changes and are most frequently overlooked by conventional methods. The methodology presented here disentangles the trends across quantiles, enabling the identification of changes across specific thresholds (see Supplementary Information and Supplementary Fig. S2).

## Typology of Precipitation Variability and Risk

**Figure 3:**
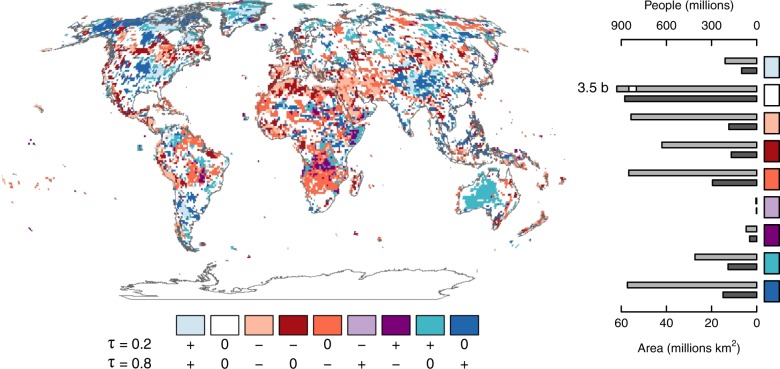
Tail typology combines dry and wet tail trends to synthesize variability and risk of precipitation excesses and deficits. All pairwise combinations of positive (+) and negative (−) significant, and non-significant (0) trends in *τ* = 0.2 (dry) and *τ* = 0.8 (wet) tails. Total land area (black) and population (gray), associated with each type are provided as a bar graph. Greenland is excluded from statistics due to sparse population.

Large deviations from mean annual precipitation can impart significant stress to human and ecological systems. Thus, simultaneous analysis of trends in the upper and lower tails of the precipitation PDF allows for assessment of the compounded influence of risk and variability (see Methods). Pairwise combinations of changes in the wet and dry tails, summarized as positive, negative, and no trend, yield 9 distinct patterns that characterize risk and variability^29^ (Fig. 3). The first three elements in the legend indicate consonant tail trends while the remaining 6 indicate sensitivity in either a single quantile or both quantiles with opposing directional responses implying changes in inter-annual variability as well as risk. We find that all regions exhibit cases of single-tail or diverging tail trends, with 38.4% of the global population and 44.4% of land area coinciding with overlooked trends. The most frequently overlooked trends include an increased risk of an extreme wet conditions (high annual total) and increased variability (positive trend in *τ* = 0.8 only) found in the Midwestern United States, Northern Canada, South-Central Asia, and Indonesia impacting 860 million people globally. Conversely, 840 million people are exposed to a decreased risk of wet conditions due to negative trends in *τ* = 0.8, particularly over Southern Africa, South America, and parts of Northern Asia, indicating a decrease in the incidence of high annual totals. An increased risk of dry conditions coincides with 630 million people (negative trends in *τ* = 0.2) in parts of southern Europe, the western United States, southern Canada, and northern Africa. Such single-tailed trends are not discernible in LR analysis, where a common trend is ascribed to all quantiles. Furthermore, changes in interannual variability are systematically parsed into 3 typologies each whereby for trend significance and direction $({\tau }_{0.2},\,{\tau }_{0.8})$: a) increased interannual variability: (0, +), (−, 0), (−, +) and b) decreased variability: (+, 0), (0, −), (+, −). We note that relatively few areas (2.4%) exhibit significant trends of opposite sign in the upper and lower quantiles, compared to the other 4 types (41.9%). As diversity of changes may entail single or two-tailed trends, QR allows for risk and interannual variability to be assessed in a detailed manner.

A related concern is with trends towards increasing and decreasing annual precipitation in the wettest and driest regions. Grids were ranked by long-term mean annual totals, and land areas corresponding to the 20% highest and 20% lowest totals were used for further analysis. The frequency of typology occurrence shows that 26.7% of the driest land area coincides with negative trends in single or both tails of the PDF, including parts of Northern Africa, the Middle East, and Central Asia. Similarly, 24.2% of the wettest area coincides with positive trends constituting a higher probability of wetter conditions, particularly over the sub-tropics including parts of South America, central Africa, and Indonesia. LR identifies fewer areas than QR, overlooking trends in the wettest and driest regions of the world (see Supplementary Fig. S3), further demonstrating the need for trend reappraisal. In total, 2.8 billion people and 58.8 million km^2^ coincide with changes overlooked by LR-based methods. Trend typology enables a detailed characterization of the nature of precipitation changes. However, we note that the level to which a trend constitutes a significant impact depends on the magnitude of the trend, context, and level of sensitivity, as a system may be more or less sensitive to changes in one tail than the other. The spatial extent and populations associated with these overlooked trends underscores their importance for understanding potential risk to populations, future water supply, and water-sensitive systems.

## Overlooked Trends in Rainfed Agricultural Systems

**Figure 4:**
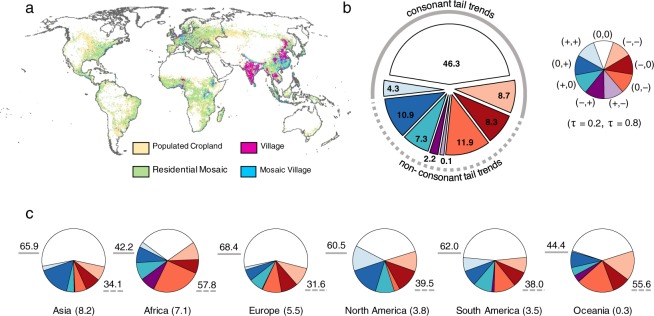
Overlooked trends coincide with regions dominated by rainfed agriculture. (**a**) spatial distribution of rainfed land types^30^. (**b**) percentage of rainfed land area coinciding with tail-trend typology. (**c**) percentage of rainfed land in each region associated with tail trends. Total rainfed area in million km^2^ is shown in parentheses. Percentage of land exposed to consonant and non-consonant tail trends are noted to the upper left and lower right of each plot respectively.

To illustrate the implications of overlooked trends on human systems, we assess trends in rainfed agriculture dominated areas^30^ (Fig. 4a). Rainfed agriculture is particularly widespread in Africa, Southeast Asia, the Midwestern United States, and Europe, constituting approximately 75% of total agricultural land (see Supplementary Table S2). We note that there are uncertainties in determining the precise location and extent of rainfed and irrigated agriculture^31^. Rainfed agriculture is more sensitive to changes in precipitation than irrigated agriculture, particularly in locations with low levels of infrastructure and water supply^32^. In regions such as Sub-Saharan Africa where there is a high reliance on rainfed agriculture to meet nutrition and economic needs, there may be higher vulnerabilities to changes in climate, demographics, and global trade^33^. By intersecting trend typology with land areas characterized by predominantly rainfed land uses, we find that 53.7% of all rainfed land area coincides with significant tail trends, with 40.7% characterized by non-consonant tail trends overlooked by LR (Fig. 4b). Crop-specific precipitation thresholds are sensitive to various aspects of precipitation (annual, seasonal, intensity, frequency), may be sensitive to other climatic variables^34^, and are used to inform management practices. For example, sorghum yields in West Africa increase with precipitation intensity and decrease with more frequent lower level precipitation in years with lower annual rainfall, while at higher annual amounts yields may decrease due to increased nitrogen soil leaching^35^. Regionally, our results show that 57.8% of rainfed area in Africa is characterized by overlooked trends, with more than half showing an increased risk of drier conditions (negative trend in *τ* = 0.2 or *τ* = 0.8) (Fig. 4c), primarily over western and southern Africa (Fig. 3). Wheat cropping practices in the United States Pacific Northwest and Midwest are also sensitive to precipitation thresholds, with lower yields associated with summer fallow – winter wheat planting practices when precipitation is high as compared to planting directly after other crops^36,37^, demonstrating the role of precipitation thresholds in determining planting strategies. We find an increased risk of wet conditions across rainfed agriculture areas in North America, notably in the Midwest region. We also find high sensitivities to overlooked trends in Australia (55.6%) and Asia (34.1%). Given the wide range of crop sensitivities to climatic variables, this illustrated approach may be generalized for assessment at any crop-specific thresholds.

## Mischaracterization of Trends Underestimates Risk to Human and Environmental Systems

Human adaptation to climate change requires understanding the likelihood of experiencing detrimental impacts. Mischaracterization of risks to human and environmental systems may underestimate the urgency of climate adaptation or could lead to inappropriate strategies. Our results show that significant population and land areas on the global scale correspond with changes in precipitation risk and variability and are mischaracterized by conventional approaches. We identify regions where trends in the lower and upper tails, of high salience for climate impacts assessment, are inconsistent with those in the mean and median. In rainfed agricultural areas - one context where precipitation variability and change inform decision-making and adaptation strategies - QR-based methodology identifies overlooked trends. Furthermore, unreliable or erroneous estimates of risk is of special concern for more vulnerable contexts and communities. Our results underscore how trends overlooked in terms of spatial extent, regionality, and severity have implications for a range of human and environmental systems. This is particularly notable given the use of LR-based methods to assess observed and future trends in precipitation; IPCC reports are one prominent example^21^. Here we offer a reappraisal of risk across thresholds in human and environmental systems, with previously undetected changes present in all regions of the globe. Future work is needed to determine if overlooked trends are replicated in general circulation models – a widely used tool for projecting climate-induced risks to earth systems –and assess how they may change in response to future climate variability. Detection of future trends across a range of thresholds allows for risk assessment at more appropriate adaptation targets.

## Methods

### Data selection and processing

#### Precipitation

Monthly mean precipitation grids at 1° × 1° resolution from June 1950–May 2017 are used for this study^25^. Grids were aggregated on a June to May annual year to better preserve a) monsoonal precipitation and b) ENSO - driven precipitation on an annual scale. For example, the designation of 1950 is defined by June 1950–May 1951. The aggregated monthly mean precipitation grids were multiplied by 365.25/12 to produce the annual total precipitation at each grid cell. To ensure commensurability in the mean trends, LR was conducted for the 1950–2016 period for both June–May and January–December annual year designations (Supplementary Fig. S4). Slope coefficients have a correlation of 0.99 on the global scale.

#### Population

Population data was obtained from the 2015 UN-adjusted population count v4 dataset^38^ at a 1 km resolution.

#### Rainfed agriculture

Rainfed agriculture land types were extracted from the Anthropogenic Biomes of the World v1^30^. Data is at a 10 km resolution. Anthropogenic biomes with the descriptor of rainfed (Rainfed Villages, Rainfed Mosaic Villages, Residential Rainfed Mosaic, and Populated Rainfed Cropland) were chosen as representative of rainfed agriculture land types. Descriptions of all land types in the dataset can be found in ref.^39^.

### Precipitation data quality control

To account for differences in results arising from the use of alternative gridded precipitation products, confirmatory analysis was pursued. We compared the PREC/L at a 0.5° × 0.5° resolution^25^ and CRU TS 4.01 monthly precipitation^40^ datasets on a June–May annual year from 1950–2011. We find that the correlation of the annual total time series is high for large areas of the globe (Supplementary Fig. S5), with low correlation over parts of North and Central Africa, upper northern latitudes, and western South America. Differences in these areas likely arise due to spare station coverage and differing interpolation processes^41^. We find that the correlation of global annual mean precipitation between the products is r = 0.95, showing high agreement. To ensure that regression results are not biased due to year-to-year persistence in the precipitation data, we applied autocorrelation^42,43^, finding significant lag 1 autocorrelation in only 7.7% of grid cells (details found in Supplementary Information and Supplementary Fig. S6).

### Trend calculation with wild bootstrap

Trends are assessed through Quantile Regression (QR)^24,44^ using the quantreg package for R^45^. This work uses a linear QR model as:

1 $$Y(\tau |x)={\beta }_{0}^{(\tau )}+{\beta }_{1}^{(\tau )}x+{{\epsilon }}^{(\tau )}$$

where Y is annual precipitation, *τ* is the quantile such that 0 < *τ* < 1, ${\beta }_{0}^{(\tau )}$ is the intercept, ${\beta }_{1}^{(\tau )}$ is the slope, x is the time-series 1950–2016, and ${{\epsilon }}^{(\tau )}$ are the errors. QR allows for the conditional estimation of the precipitation level at quantile *τ*, such that $(1-\tau )$ proportion of the historical data is greater than the evaluated precipitation level. A no-cross restriction^46^ is placed on the calculation of ${\beta }_{0}^{(\tau )}$ and ${\beta }_{1}^{(\tau )}$ such that the regression lines calculated at each quantile do not intersect, which would produce physically indefensible results. The time series 1950–2016 is standardized and centered on 0 prior to data fitting, as large ranges of covariate values can lead to erroneous intercept calculations^47^. Trends are assessed at the median (*τ* = 0.5) as a robust measure of central tendency, as it is insensitive to outliers, and at *τ* = 0.2 and 0.8 representing the dry and wet tails respectively. Wild bootstrap^48^ is used to measure significance as it accounts for heteroscedasticity in the residuals. Residuals were resampled, weighted, and the fit recalculated n = 1000 times with a significance level of 𝛼 = 0.05. Residuals at each grid point were sampled in the same order to minimize differences between neighboring cells.

### Trend typology

To capture the diversity of changes in the tails of the precipitation probability distribution function, pairwise combinations of positive and negative significant, and non-significant slope coefficients for ${\beta }_{1}^{({\tau }_{1})}$ and ${\beta }_{1}^{({\tau }_{2})}$ are evaluated for *τ* = 0.2 and 0.8. There are 9 unique combinations of positive and negative changes between the upper and lower quantile where at the response for at least one quantile is significant. Typologies where the response in only one quantile is significant implies changes variability. To assess how overlooked trends coincide with population, typology grids were first resampled to a 1 km resolution and population grids were aggregated by typology using ArcGIS 10.2. Typology grids were similarly resampled to a 10 km resolution prior to intersection with the rainfed land types to determine the percentage of rainfed area characterized by each typology.

## Electronic supplementary material

Supplemental Material
