# Supplementary material for: Overlooked Trends in Observed Global Annual Precipitation Reveal Underestimated Risks
Source: Sci Rep. 2018 Nov 13;8:16746. doi: 10.1038/s41598-018-34993-5 (PMC6233197; doi:10.1038/s41598-018-34993-5)
Supplement: Supplementary file 1 — Supplemental Material [file 41598_2018_34993_MOESM1_ESM.docx]

Supplemental Material

**Overlooked Trends in Observed Global Annual Precipitation Reveal Underestimated Risks**

Anne M. Lausier1* and Shaleen Jain1
1Department of Civil and Environmental Engineering, University of Maine, Orono, ME, USA. *e-mail: anne.lausier@maine.edu

Selection of quantiles to represent precipitation tail responses

The conditional quantile response can be evaluated for any 𝜏 where 0 < 𝜏 < 1, providing flexibility for evaluating changes over the range of outcomes and thresholds in human and environmental systems. The entire precipitation probability distribution function (PDF) can be estimated using quantile regression (QR). For this study we chose 𝜏 = 0.2 and 𝜏 = 0.8 to represent the annual precipitation tails. For annual precipitation data, 𝜏 = 0.2 refers to the precipitation total that is less than 80% of the historical annual totals, where 𝜏 = 0.8 is less than 20% of the recorded totals. To demonstrate the range of trend estimations achieved by the QR model, and how linear regression (LR) mischaracterizes the quantile response across precipitation levels, we select two grid locations in the US for comparison (Fig. S1). For each location, the annual precipitation data is evaluated for all 0 < 𝜏 < 1 at 0.1 intervals. Significance is tested using the wild bootstrap method with n = 1000 (see methods for more details on wild bootstrap). For the location marked in blue, there is a significant mean trend. LR assumptions imply that the trend in the mean is the trend across all quantiles with the entire distribution showing a shift in location without a change in variance. The quantile coefficient estimations show that only 𝜏 > 0.4 is significantly changing with the coefficients close to 0 mm/yr in the lower quantiles. Thus, LR underestimates the risk of dry conditions. Conversely, the location marked in green shows a significant positive response for most 𝜏 < 0.5 and largely non-significant changes in the upper tail. LR estimates an increase in the position of all quantiles, thereby overestimating the risk of wet conditions. Analysis of these two grids help to highlight the range of sensitivities across quantiles. We chose 𝜏 = 0.2 and 𝜏 = 0.8 as thresholds to represent the tails due to sample size. QR places higher weights on the points closest to the specified quantile, resulting in very high and low 𝜏 being sensitive to few data points. With a sample size of 67 years, the coefficient estimations for quantiles 𝜏 < 0.2 and 𝜏 > 0.8 tend to have higher errors producing unreliable estimates. We find that the coefficient estimates at 𝜏 = 0.2 and 𝜏 = 0.8 are similar to estimates in more extreme quantiles, and often are even conservative estimates of changes in the tails. With a larger sample size, higher and lower quantiles could be selected due to lower estimation errors.

Assessment of Changes at Specified Thresholds

For the selected grid location in the upper panel of Fig. S1, the conditional quantile regression lines and mean trend show marked asymmetric changes over time (Fig. S2). A comparison of the regression lines clearly demonstrates that the slope coefficients of 𝜏 = 0.2 and 0.8 are unequal, and that variability in year to year precipitation totals has increased over time at this location. Only 𝜏 = 0.5 and 0.8 show significant changes, while 𝜏 = 0.2 shows low magnitude non-significant change (0.3 mm/year). LR assumes that the precipitation PDF has constant variance and that the entire distribution undergoes a location-shift with the conditional mean. Thus, at this location LR will underestimate precipitation variability, and underestimate the risk of experiencing a high precipitation total. As noted in Fig. S1, QR can be used to evaluate changes at any threshold in the distribution and can characterize the entire range of precipitation variability over time.

Trend typologies in driest and wettest regions of the world

The frequency of trend typologies coinciding with the long-term annual mean precipitation totals show the nature of change for dry and wet regions of the globes (Fig. S3). Annual mean precipitation totals grids were ranked and placed in quantile bins subdividing the globe into very dry to very wet regions. The frequency of typologies falling in each bin were identified, as were the frequency of positive, negative, and non-significant mean trends. Figure S3 indicates that the driest and wettest regions of the globe are characterized by higher frequencies of significant trends than the middle 20%. In both the driest ([0, 0.2)) and wettest ([0, 0.8)) regions of the globe approximately 25% of each region is characterized by negative and positive trends in single or both tails respectively. This reflects increasing differences between wet and dry areas. However, increased probability of wet conditions in dry areas and drying in wet areas are evident as well. Comparison with LR shows that the mean trends follow a similar pattern of positive and negative trends but underestimate the affected area. As such, QR detects overlooked trends in all regions of the globe, but particularly in the wetter and drier areas where sensitivities to change may be higher.

Autocorrelation of Annual Precipitation Totals

An underlying assumption of statistical methods for assessing climatic trends is that observations are independent, and the time-series is stationary. Violations of these assumptions can lead to erroneous trend significance calculations. To ensure that QR trend results are not biased due to year-to-year persistence in the precipitation data we apply autocorrelation^1^ defined by:

$$r\left( k \right)= \frac{\sum_{i}^{N-k} (Y_{i}-\bar{Y})(Y_{i+k}-\bar{Y})}{\sum_{i=1}^{N} {(Y_{i}-\bar{Y})}^{2}} \left( 1 \right)$$

Where r(k) is the autocorrelation at lag k, N is the number of observations, and Y1, Y2, Y3,. . . YN are observations at equally spaced time intervals. We compare the autocorrelation of PREC/L at a 0.5° x 0.5° resolution^2^ and CRU TS 4.01 monthly precipitation^3^ datasets on a June – May annual year from 1950 – 2011. Following a procedure used for other annual precipitation total time-series^4^, we first screened the data based on the number of gauges present in each 1 x 1 grid cell^5^. Autocorrelation was only performed on grid cells with at least 1 gauge present in the 1950—2016 record (Fig. S6). The distribution of autocorrelation follows a normal distribution, with the 90% and 95% confidence intervals evaluated at $\pm\frac{1.65}{\sqrt{n}}$ and $\pm\frac{2.0}{\sqrt{n}}$ respectively, where *n* is 67 years. We find that 7.7% of all land area grids show significant lag 1 autocorrelation at a 95% confidence interval. The impacts of episodic events on precipitation persistence are minimized by selecting a June - May annual year designation that better captures monsoonal precipitation as well as evolving ENSO conditions that often initiate and mature over the summer- fall-winter period.


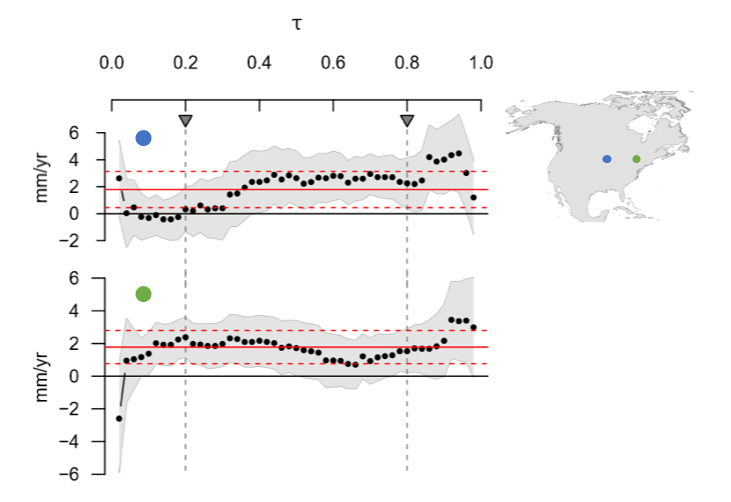


**Figure S1.** Linear Quantile Regression coefficient estimates over the full range of quantiles (𝜏) for two sample locations in North America. Gray shaded areas show the 90% confidence interval (CI) with significance determined using wild bootstrap (*n* = 1000). The solid red line shows the mean trend with dashed lines showing the 90% CI. Locations of 𝜏 = 0.2 and 0.8 are marked. In both examples, the mean trend over or underestimates the trend at different parts of the distribution.


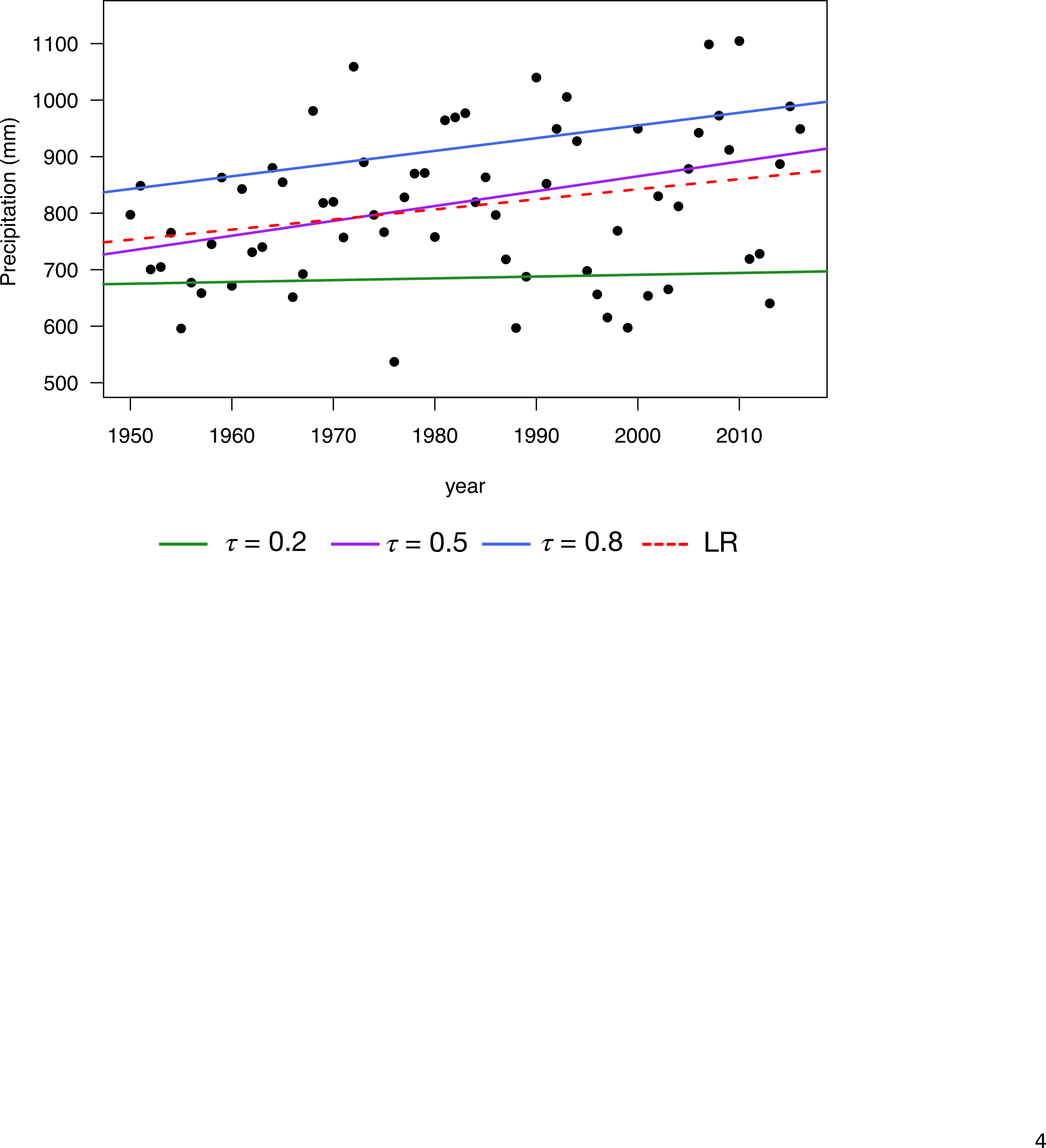


**Figure S2.** LR mischaracterizes risk at thresholds in the upper and lower tails of the annual precipitation distribution. Conditional trend estimates at the blue marked location in Supplementary Figure 1. LR, 𝜏 = 0.5 and 0.8 are all significant at 𝛼 = 0.05. QR can be used to estimate the trend at any threshold of the precipitation distribution, characterizing the entire range of variability.

**
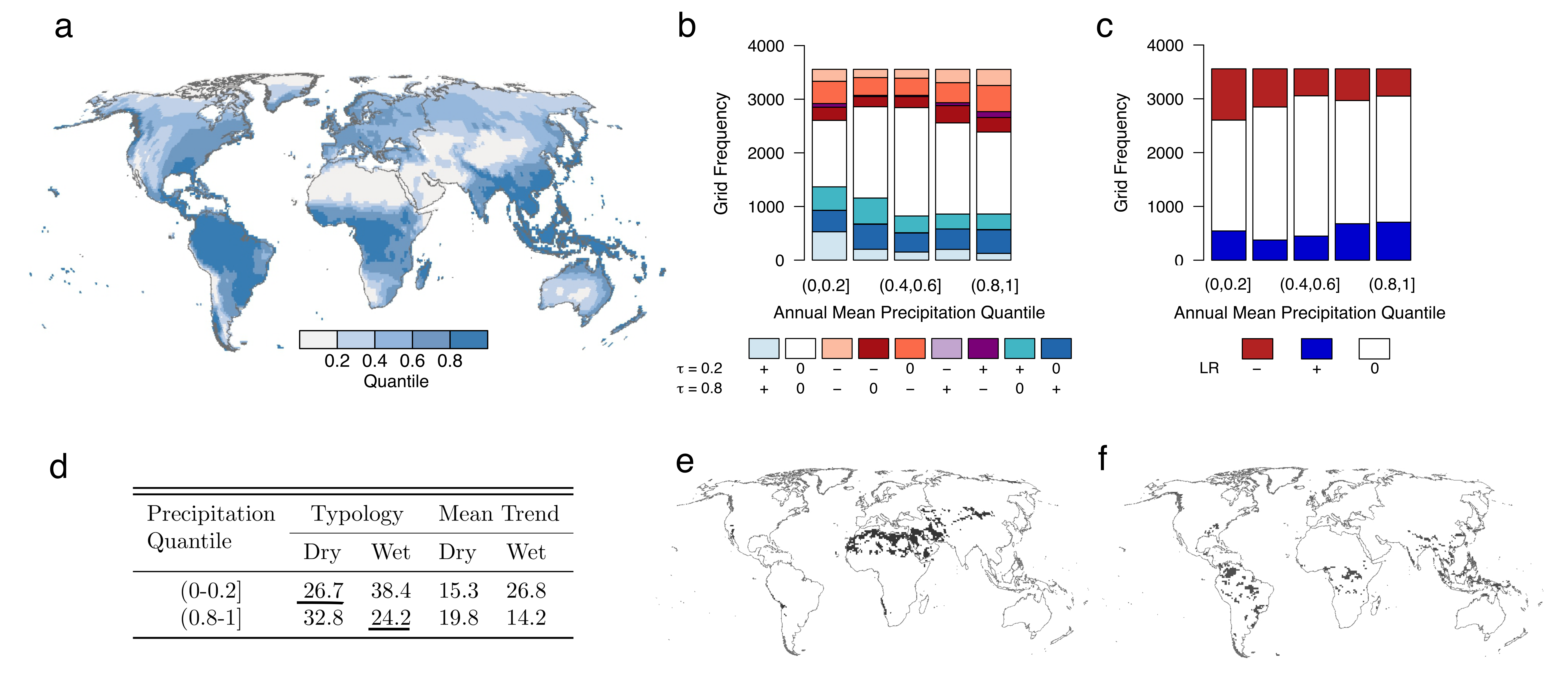
Figure S3**. Assessment of trends in annual precipitation conditioned on the long-term mean precipitation totals. a) Global pattern of mean annual precipitation, expressed as quantiles. b-c) Frequency of typology and mean trend across quantile ranges. d) Area in the driest 20% and wettest 20% of global regions coinciding with typologies and mean trends showing increased risk of wet and dry conditions. Underlined values indicate dry areas with increased risk of dry conditions, and wet with wetter conditions. e) driest regions undergoing decreases in precipitation and f) wettest regions getting wetter. Greenland excluded from analysis.


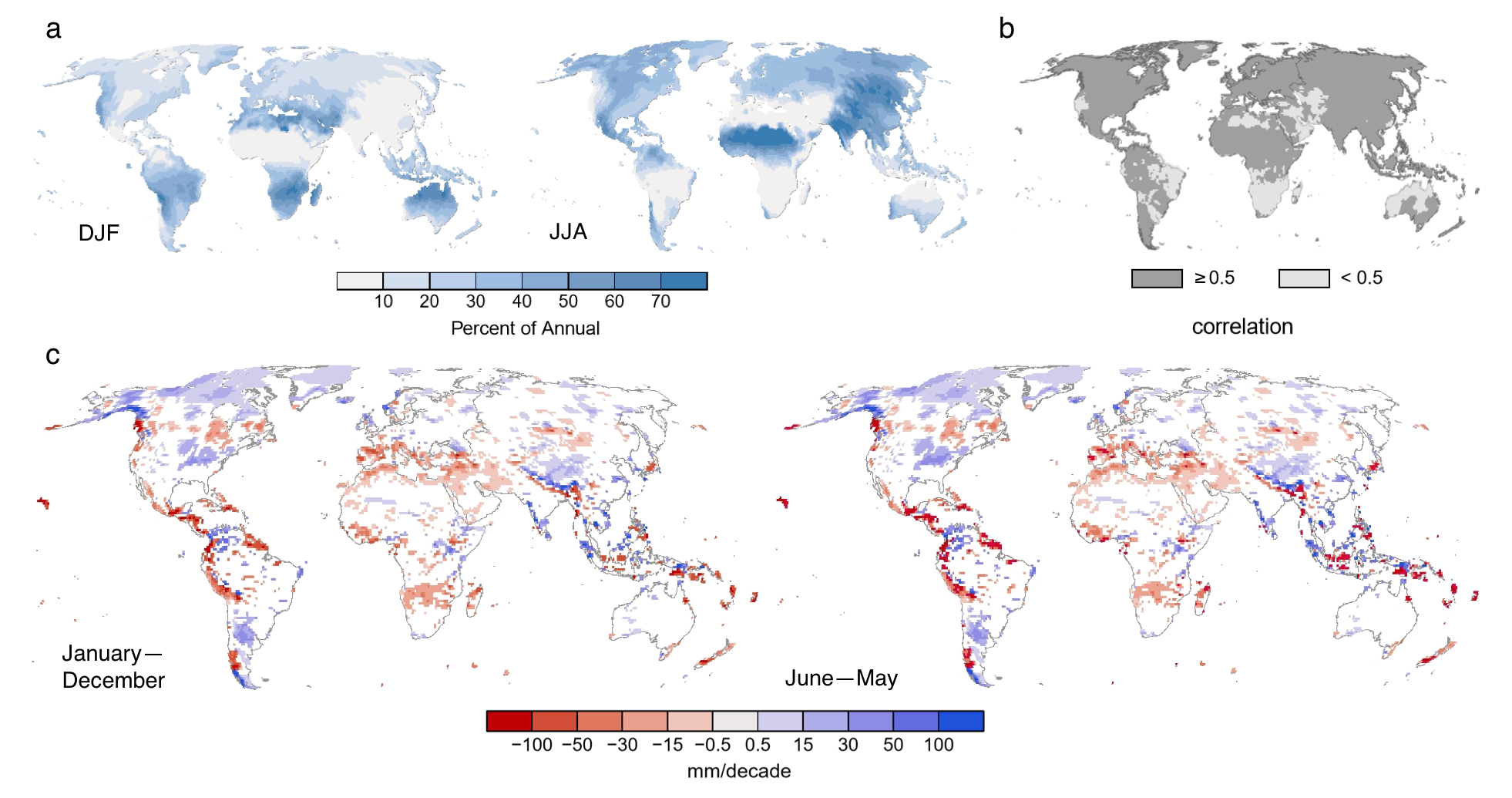


Figure S4. Annual Definition Impacts Annual Totals. a) Average percent contribution of winter and summer seasons to annual precipitation. b) correlation of annual totals using a January—December and June—May annual definitions. For locations with high percentages of annual rainfall in DJF, the correlation is poor. c) Annual mean trend for 1950—2016 using January—December and June—May annual definitions. The correlation between regression coefficients is r = 0.99. Change in annual definition does not significantly impact mean trend.


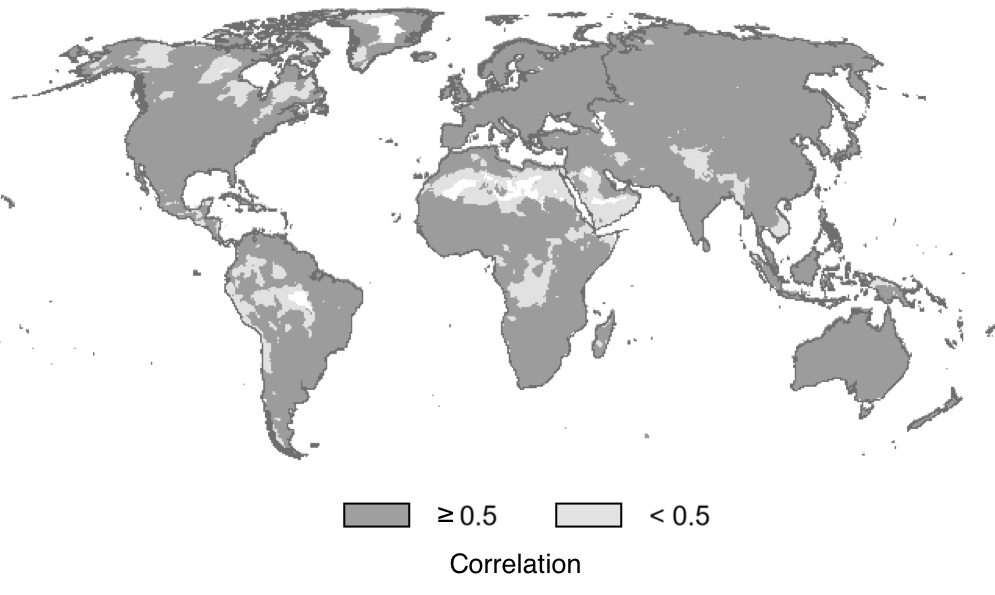


**Figure S5.** Correlation coefficient of annual precipitation time-series (1950 – 2011) NOAA PREC/L and CRU TS 4.01 datasets at 0.5º x 0.5º resolution. Correlation of mean annual precipitation between the two datasets is r = 0.95


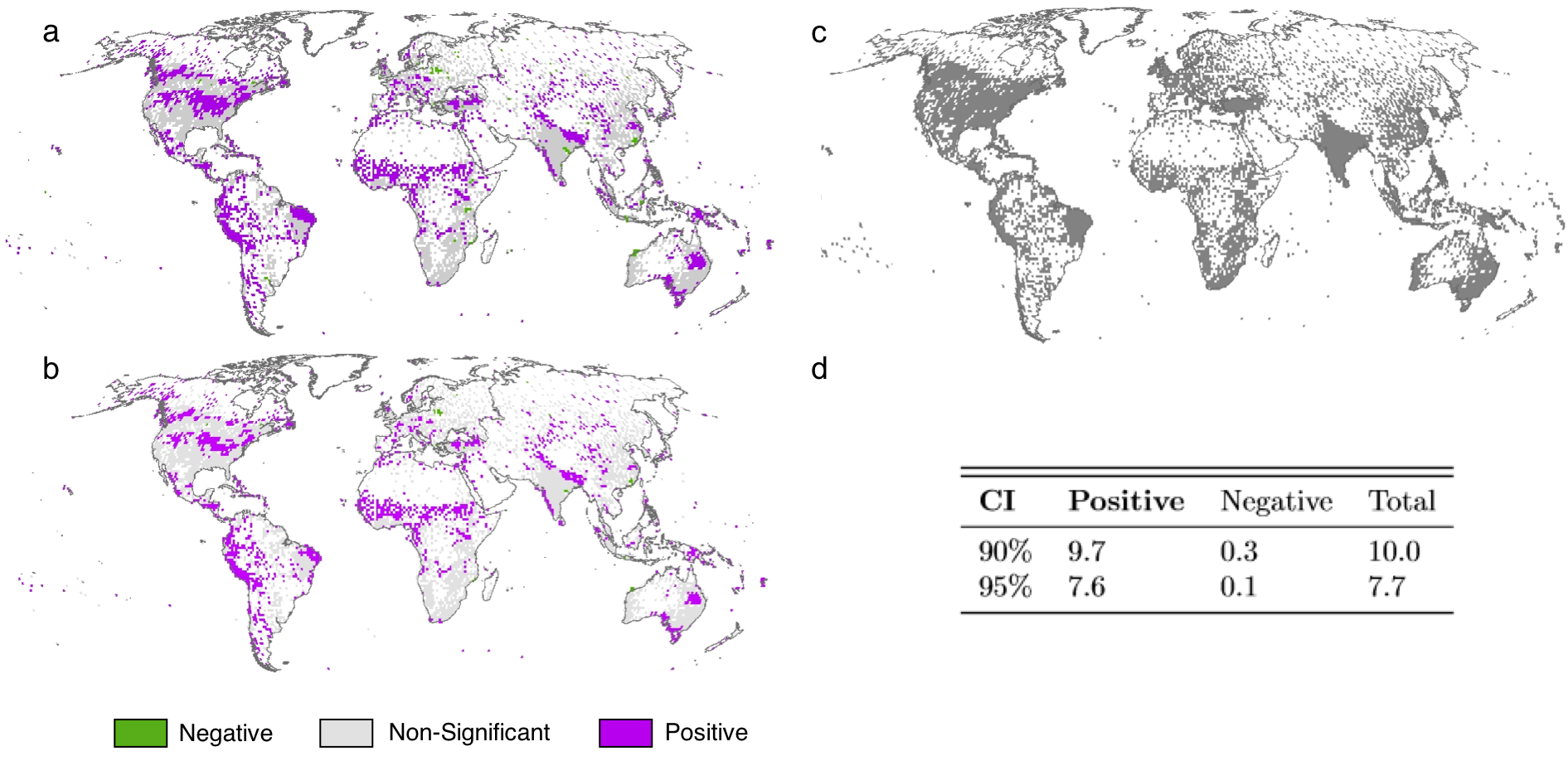


**Figure S6**. a) Autocorrelation of annual precipitation (1950 – 2016) at lag(1) at a 90% confidence interval (± 0.2). b) lag (1) autocorrelation at a 95% confidence interval (± 0.244). C) Autocorrelation is only evaluated for grids with at least 1 station (27.0% of total grids) D) The percentage of land area grids (excluding Antarctica) with significant lag 1 autocorrelation at 90% and 95% confidence intervals (CI).


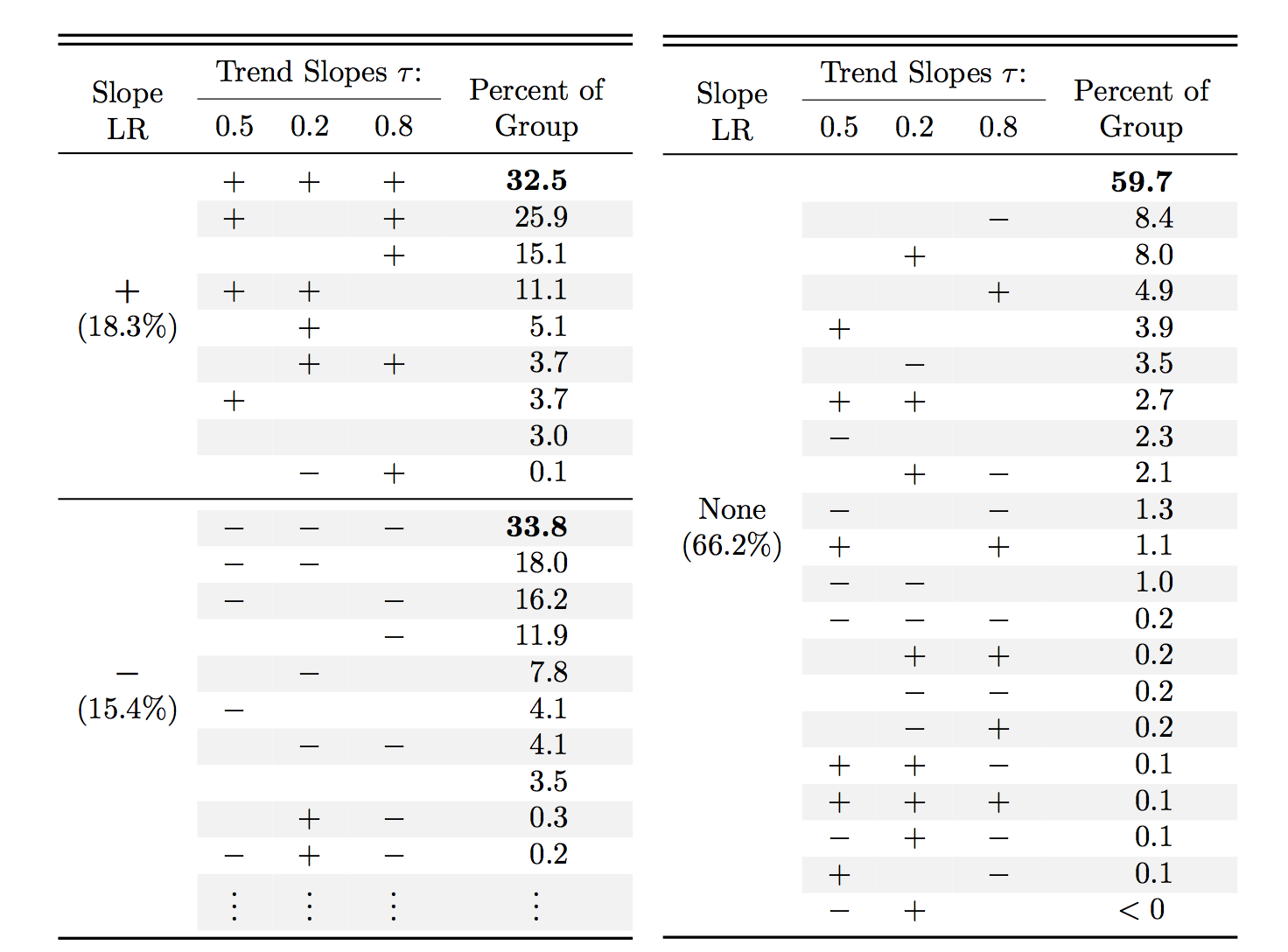


Table S1. Global significant precipitation trend patterns across quantiles for positive, negative, and non-significant trends in LR. + and – indicate positive and negative significant responses. Blank spaces indicate no significant response. Percent of grids contributing the positive, negative, and non-significant slope coefficients from LR are shown in parentheses. Percent of Group column refers to the percentage of grids in each LR sub-group. Trends are significant at 𝛼 = 0.05.


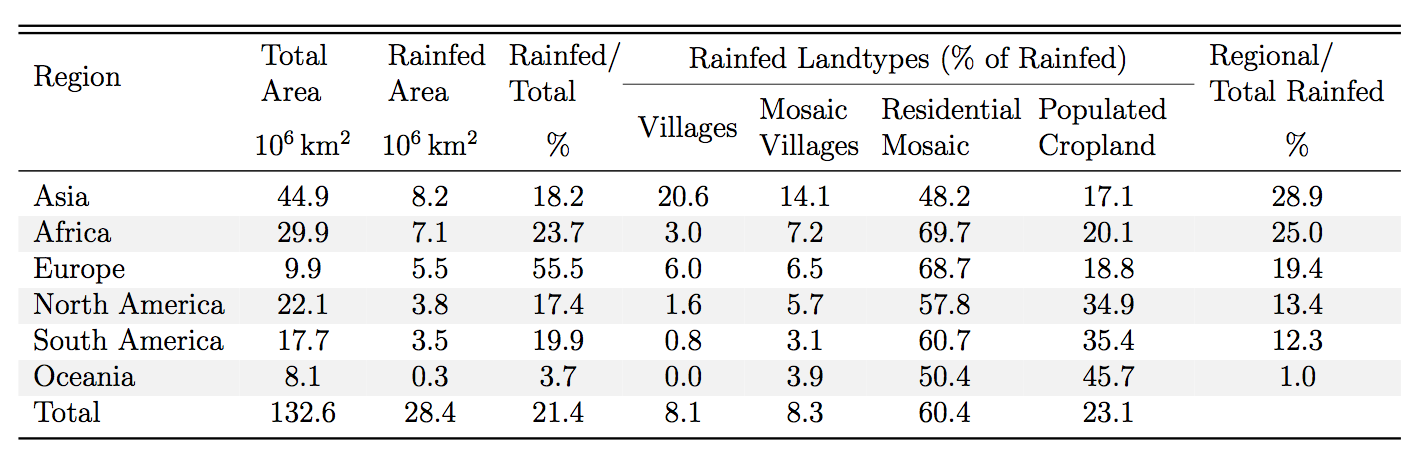


Table S2. Rainfed land types across regions. Percentage of regional area constituted by rainfed land types sensitive to precipitation. 21.4% of total land area excluding Antarctica and Greenland is comprised of rainfed land types.

**References**

1. Box, G. E. P., Jenkins, G. M., Reinsel, G. C. & Ljung, G. M. *Time series analysis: Forecasting and control* 5^th^ edn (John Wiley & Sons Inc., New York, 2015).
2. NOAA Precipitation Reconstruction over Land (PREC/L) dataset (Earth System Research Laboratory Physical Sciences Division, accessed 2 August 2017) https://www.esrl.noaa.gov/psd/data/gridded/data.precl.html
3. Harris, I.C.; Jones, P.D. CRU TS 4.01: Climatic Research Unit (CRU) Time-Series (TS) version 4.01 of high-resolution gridded data of month-by-month variation in climate (Jan. 1901- Dec. 2016) (Centre for Environmental Data Analysis, accessed 1 March 2018), http://dx.doi.org/10.5285/58a8802721c94c66ae45c3baa4d814d0
4. Sun, F., Roderick, M. L. & Farquhar, G. D. Rainfall statistics, stationarity, and climate change. *Proc. Natl. Acad. Sci. U.S.A.* **115,** 2305-2310 (2018).
5. Chen, M., Xie, P., Janowiak, J. E. & Arkin, P. A. Global Land Precipitation: A 50-yr Monthly Analysis Based on Gauge Observations. *J. of Hydrometeorol.* **3,** 249-266 (2002).
